# Application of Proteomics and Peptidomics to COPD

**DOI:** 10.1155/2014/764581

**Published:** 2014-05-05

**Authors:** Girolamo Pelaia, Rosa Terracciano, Alessandro Vatrella, Luca Gallelli, Maria Teresa Busceti, Cecilia Calabrese, Cristiana Stellato, Rocco Savino, Rosario Maselli

**Affiliations:** ^1^Department of Medical and Surgical Sciences, University “Magna Græcia” of Catanzaro, Catanzaro, Italy; ^2^Department of Health Science, University “Magna Græcia” of Catanzaro, Catanzaro, Italy; ^3^Department of Medicine and Surgery, University of Salerno, Salerno, Italy; ^4^Department of Respiratory Medicine, Second University of Naples, Caserta, Italy

## Abstract

Chronic obstructive pulmonary disease (COPD) is a complex disorder involving both airways and lung parenchyma, usually associated with progressive and poorly reversible airflow limitation. In order to better characterize the phenotypic heterogeneity and the prognosis of patients with COPD, there is currently an urgent need for discovery and validation of reliable disease biomarkers. Within this context, proteomic and peptidomic techniques are emerging as very valuable tools that can be applied to both systemic and pulmonary samples, including peripheral blood, induced sputum, exhaled breath condensate, bronchoalveolar lavage fluid, and lung tissues. Identification of COPD biomarkers by means of proteomic and peptidomic approaches can thus also lead to discovery of new molecular targets potentially useful to improve and personalize the therapeutic management of this widespread respiratory disease.

## 1. Introduction

Chronic obstructive pulmonary disease (COPD) is a widespread respiratory disorder involving both airways and lung parenchyma, usually characterized by progressive and poorly reversible airflow limitation and largely caused by tobacco smoke and air pollutants. Nowadays, there is a continuous increase in the prevalence of this common disease, which affects more than 200 million patients mainly including elder people, and current epidemiological projections foresee that by 2020 COPD will become the third leading cause of death worldwide [1]. However, despite the intense research efforts involving huge human and economic resources, the cellular and molecular mechanisms underlying COPD pathobiology are not yet well understood. Furthermore, it is clearly evident that COPD is a heterogeneous disease characterized by several different phenotypes, distinguishable with respect to clinical presentation, frequency of exacerbations, pathophysiologic aspects, rate of lung function decline, radiologic imaging, and response to treatment [2]. Therefore, there is an urgent need to improve our knowledge of the inflammatory, immune, and structural substrates of the various COPD phenotypes, which should be ideally identifiable on the basis of expression of specific disease biomarkers. In this regard, a significant contribution could be provided by translational bench-to-bedside (and back again) approaches, also including the application of proteomic and peptidomic techniques to biological samples obtained from distinct subgroups of COPD patients [3]. Indeed, in spite of the valuable importance of genomic and transcriptomic studies, a single gene or a single mature messenger RNA (mRNA) can be associated with several different proteins and peptides due to RNA splicing and editing, as well as to posttranslational modifications [4]. This implies that mRNA cellular concentrations can even be poorly correlated with protein levels, which are essentially responsible for biologic functions and for the expression of physiologic and pathologic phenotypes. Moreover, acellular compartments such as plasma and lung epithelial lining fluid (ELF) have small quantities of DNA or RNA but may have large amounts of proteins that could be relevant disease biomarkers [4]. Within such a context, the aim of this review is to provide an overview of both methods and samples used to investigate the proteomic and peptidomic profiles of patients with COPD. In particular, our discussion will focus on currently available information obtained by sampling and studying peripheral blood, induced sputum, exhaled breath condensate, bronchoalveolar lavage fluid, and lung biopsies.

## 2. Proteomic Strategies: An Outline

In every body fluid or sample such as plasma or serum, the overall protein content can be assayed and qualified via sequential technical steps, starting from protein separation. For proteomic analysis, protein separation is usually performed by two-dimensional electrophoresis (2-DE), based on a pH gradient (first dimension) within an electric field, where protein migration is dependent on the isoelectric point. The second dimension depends on protein size (molecular weight) that is responsible for migration speed through the electrophoretic field of a dodecyl sulfate polyacrylamide gel (PAGE). Proteins can be then excised from the gel, denatured to their primary, linear structure, and digested with a protease such as trypsin, which produces predictable protein fragments [4]. Protein fractionation can also be performed via chromatographic separation, based on protein affinity for chemical substances. The most used chromatographic method is reverse-phase liquid chromatography, based on the concept that hydrophobic proteins and peptides will elute from hydrophobic columns at progressively higher concentrations of organic solvents [4].

After fractionation and proteolytic digestion, protein identification mostly relies on mass spectrometry (MS), based on separation of gaseous ions according to their different mass and charge [5]. Therefore, MS can be used to identify and qualify peptides as well as large and small proteins. In order to generate charged molecules, the most commonly used ionization method is matrix-assisted laser-desorption ionization (MALDI), which is often coupled to “time-of-flight” (TOF) analyzers (MALDI-TOF) [6]. Indeed, MALDI-TOF-based profiling can be a very useful tool for biomarker discovery because it provides a direct analysis of low-molecular-weight metabolites, endogenous peptides, and proteins in bodily fluids. Specifically, the comparative analysis of molecular signatures from enriched or fractionated subproteomes from healthy/diseased or drug treated/untreated biosamples, based on MALDI-TOF or its variant surface-enhanced laser-desorption/ionization time-of-flight mass spectrometry (SELDI-TOF-MS), allows speed of analysis as well as sensitivity and tolerance against salts and detergents present in biological samples. Because of the large dataset generated by MS spectra, mathematical algorithms need to be applied with the aim of recognizing biomarker patterns suitable for distinguishing both classes. When a given pattern displays significant differences between the diseased and normal samples, identification of individual discriminatory peaks is performed by MALDI-MS/MS analysis [6].

Therefore, we can summarize that proteomic-based strategies are largely dominated by two-dimensional polyacrylamide gel electrophoresis (2D-PAGE, also known as 2D-GEL) or liquid chromatography (LC) coupled to MS or tandem MS and MALDI-TOF -MS (or its variant SELDI-TOF-MS). The latter is mainly finalized to target a restricted window of the proteome, consisting of peptides and small proteins not easily manageable by conventional gel electrophoresis.

## 3. Blood 

Analysis of plasma proteome may contribute to identifying interesting COPD biomarkers. For example, 2-DE and MALDI-TOF technologies have been used to delineate the plasma proteomic profiles of three different people groups, represented by healthy control subjects, patients with COPD, and individuals with asthma, respectively [7]. The different expression patterns of a panel of four biomarkers, including *α*2-macroglobulin, haptoglobin, ceruloplasmin, and hemopexin, were able to significantly discriminate the three study groups. These data suggest that proteins involved in iron metabolism pathways and acute-phase responses can be implicated in the pathogenesis of chronic obstructive respiratory diseases [7]. Microarray-based proteomics has also been utilized to discover serum biomarkers eventually correlated with clinical aspects of COPD [8]. Indeed, using such a protein microarray platform, it has been found that expression of some proteins implicated in inflammation and tissue remodeling, including interleukin-8 and matrix metalloproteinase-9, was significantly correlated with clinical parameters and exacerbation frequency [8]. Moreover, SELDI-TOF approach has been very useful in order to characterize serum amyloid A as a sensitive biomarker of acute COPD exacerbations [9]. High-resolution reverse-phase liquid chromatography coupled to MS can help to distinguish COPD patients characterized by either a fast or a slow decline in lung function, assessed by evaluating the annualized progressive reduction of FEV_1_ (forced expiratory volume in one second) [10]. In particular, plasma proteomic analysis has made it possible to differentiate such two distinct functional phenotypes on the basis of the different expression of 55 peptides, mapped to 33 unique proteins. Some of these proteins are involved in complement or coagulation cascades [10]; however, further studies are needed to establish if overexpression of such molecules is a cause or a consequence of the different rate of lung function decline.

The main problem related to the application of proteomic/peptidomic analysis to blood in COPD arises from the very high abundance of proteins and peptides detectable in this body fluid, which hinders the attempts of really identifying suitable biomarkers directly correlated to COPD pathobiology.

## 4. Induced Sputum

Sputum is a complex secretion originating from both proximal and distal airways, which can be induced by inhalation of hypertonic saline. Induced sputum (IS) provides information about both inflammatory cells and mediators present in the airways, potentially relevant for phenotypic characterization of patients with chronic respiratory disorders such as COPD and asthma [11]. Due to the rapid development of MS, its improved accuracy and high throughput of the coupled analytical assessments can offer direct tools to evaluate the proteomic profiles of IS in inflammatory respiratory diseases. Indeed, noninvasive and reliable biomarkers detectable in IS, suitable to outline a quick disease readout, are greatly needed to speed up proof-of-concept studies in patients with COPD. Surprisingly, not so many proteomics studies referring to this biological fluid have been published so far, maybe because of the difficulties to obtain healthy control samples as well as the lack of general standardization [12]. Another reason can be the presence in sputum of highly charged mucins, which make the separation of sputum proteins via conventional techniques, such as 2-DE, quite difficult as they readily crosslink via their sulphydryl groups and hence decrease gel resolution.

However, to overcome the “mucins” problem, Nicholas et al. combined a 2D gel proteomics approach with GeLC-MS/MS, thus identifying more than 250 proteins in human sputum and demonstrating that proteomics can be applied to IS [13]. Recently, the same group used a combined platform in which proteomics (2-DGE and tandem MS) and immunoassays (Western blotting and ELISA) were applied to compare the proteins detected in IS from GOLD stage 2 COPD patients with healthy smokers. Fifteen differentially expressed protein species, identified by tandem MS over more than 1000 spots, were subsequently validated by immunoassays; in particular, lipocalin and apolipoprotein A1 were found to be significantly reduced in COPD patients when compared with healthy smokers, and their levels correlated with FEV_1_/FVC (forced vital capacity) ratio, thus demonstrating their relationship to disease severity [14]. A further effort in this direction has been recently made also by Ohlmeier et al., who identified important proteins involved in COPD pathogenesis via IS analysis performed by cysteine-specific two-dimensional difference gel electrophoresis (2D-DIGE) coupled with MS. Interestingly, by comparing IS samples obtained from nonsmokers, smokers, and smokers with moderate COPD, these authors detected in both smokers and COPD patients high levels of the polymeric immunoglobulin receptor (PIGR), a protein involved in inflammation and immune defense. PIGR was further validated by Western blotting, immunohistochemistry/image analysis, and/or ELISA in sputum, lung tissue, and blood [15]. In particular, plasma concentrations of this potential biomarker were found to be enhanced in both healthy and COPD smokers. With regard to lung specimens, high PIGR levels were expressed in bronchial and alveolar epithelium of smokers, and further PIGR increases were detected in the alveolar area of subjects with mild-to-moderate COPD [15].

Switching from 2-DE-MS- to LC-MS-based strategies, Casado et al. tried to differentiate, on the basis of distinct COPD phenotypic features and disease severity stages, the expression of more than 200 identified proteins by CapLC-ESI-Q-TOF technology [16]. Applying SELDI-TOF methodology to IS from patients with chronic inflammatory and suppurative airway diseases, Gray et al. identified potential biomarkers such as calgranulins and Clara cell secretory protein that differentiated various disease entities (COPD, asthma, cystic fibrosis, and bronchiectasis) from healthy control subjects [17].

**Figure 1:**
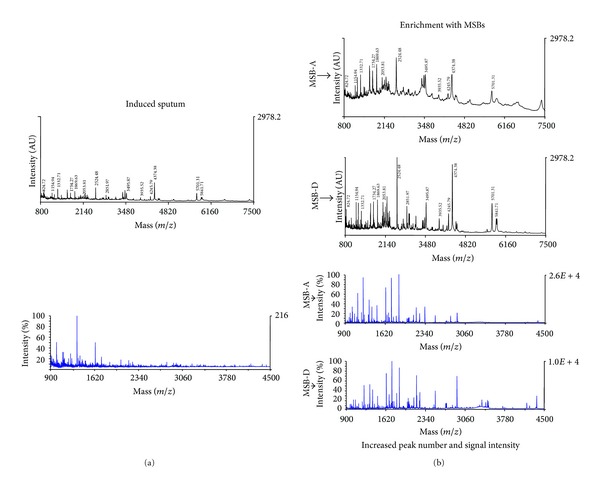
Peptidomic analysis of induced sputum: MALDI-TOF mass spectra before and after mesoporous silica beads (MSBs) processing. Utilization of MSBs makes it possible to markedly increase both peak number and signal intensity (AU: arbitrary units), referring to peptide profiles detectable in induced sputum (IS);* m/z* (mass-to-charge ratio) ranges from 800 to 7500 are reported. Average* m/z* values are indicated for selected peaks. The left side shows spectra obtained without MSB enrichment. Lower panels show MALDI-TOF profiles of the same IS sample, processed by different MSBs (MSB-A and MSB-D). Difference in surface functionalization (MSB-A versus MSB-D) gives rise to a fine modulation of the MALDI-TOF profiles.

Given the type of the abovementioned studies, it is quite evident that the low-molecular-weight components of IS are still underrepresented, and new approaches and fractionation techniques are required to extract and to characterize IS peptidome. MS profiling of peptidome for disease-associated patterns is a relatively new concept in clinical diagnostics. In this regard, peptidomic-based strategies have been used to discover disease biomarkers in a wide range of disorders, with a special focus on cancer [26]. Peptidomics defines a new promising “omics” approach for the quali-quantitative analysis of endogenous peptides in biological samples, largely by means of chromatography and other related techniques coupled to MS [27, 28]. We have recently described a peptidomic strategy based on mesoporous silica beads (MSBs) and MALDI-TOF-MS for selective binding and enrichment of plasma low-molecular-weight proteins (LMWP) [29], which has been extended also to IS (Figure 1) [18]. Based on a molecular cutoff mechanism, we used MSBs as sponges to “capture” peptides from human bodily fluids, including plasma, IS, saliva, urine, and synovial fluid. In fact, only small size molecules such as peptides or metabolites which are able to diffuse into the mesoporous channels (nanometric size) of the beads are harvested, whereas larger size proteins are excluded from the adsorptive process. Moreover, given the chemical functionality present on the mesoporous surfaces, a fine tuning of the peptidome repertoire extracted from a specific bodily fluid is also possible, thus providing rich fingerprints, which are at the basis of biomarker discovery [30]. The peptides selected and enriched by our developed MSBs could be part of a possible biomarker signature in the “sputum” of COPD patients. Indeed, MSB-MALDI-MS approach can be able to establish differential peptide profiles when comparing healthy subjects versus patients with either COPD or asthma. We found that, in contrast with asthmatic subjects, COPD patients exhibit high IS levels of the antimicrobic peptides *α*-defensins [18]. Defensins can be produced by neutrophils as well as by airway structural cells, and they are important components of the innate and adaptive immune response pathways, thus stimulating the production of proinflammatory cytokines from monocytes and bronchial epithelial cells as well as the expression of adhesion molecules by endothelial cells [31, 32]. Secretion of *α*-defensins into the airways can be elicited by infectious agents [33], thereby behaving as potential biomarkers of acute exacerbations of COPD. Furthermore, these peptides are also implicated in the recruitment, proliferation, and maturation of T lymphocytes [34].

Therefore, high-throughput screening MS-based platforms can be usefully utilized to detect key peptide biomarkers in IS samples obtained from COPD patients. However, potential drawbacks related to the use of such a source for proteomic/peptidomic purposes include contamination by saliva and the presence of substances recovered from lower airways, which sometimes make it difficult to interpret the data obtained from analyzing this sample. These problems can thus reduce the relevance of IS for periodical monitoring of COPD patients.

## 5. Exhaled Breath Condensate

Exhaled breath condensate (EBC), obtained by cooling exhaled breath collected through a noninvasive procedure, is currently considered as a suitable source of COPD biomarkers present in the lower respiratory tract [35, 36]. However, only very few data have been published with regard to this subject. Noticeably, almost all the reports published so far suggest that EBC protein levels in smokers are higher than in nonsmokers, but the clinical and biological significance of these observations is not yet known [35]. Within such a scanty context, it is particularly interesting the study performed by Fumagalli et al., who investigated the EBC proteome of COPD patients carrying genetically determined variants of pulmonary emphysema associated with *α*_1_-antitrypsin (*α*_1_-AT) deficiency, compared with healthy controls [19]. By using a proteomic research strategy based on combination of 2-DE, high pressure LC, and MS, these authors showed that *α*_1_-AT-deficient, emphysematous patients were characterized by higher EBC levels of some cytokines (IFN-*γ*, IL-2, and IL-15) and cytokeratins (CK-1, CK-9, and CK-10) than control [19]. Therefore, these molecules could represent potential biomarkers of lung injury. More recently, using liquid chromatography-tandem mass spectrometry (LC-MS/MS) and SELDI-MS, the same group has profiled the EBC proteome in nonsmokers, healthy smokers, COPD patients without emphysema, and subjects with pulmonary emphysema associated with *α*_1_-AT deficiency [20]. The results of this research approach showed that several different inflammatory cytokines, type I and II cytokeratins, two surfactant protein A isoforms, calgranulins A and B, and other proteins, could be promising indicators of the phenotypic expression of distinct COPD variants [20]. Of course, many more studies are further needed to understand whether EBC can be usefully utilized and standardized for proteomic investigations referring to COPD patients.

## 6. Bronchoalveolar Lavage

In contrast with collection of blood, IS, and EBC, bronchoalveolar lavage fluid (BALF) is recovered through an invasive procedure requiring bronchoscopic manoeuvres. Despite these limitations, SELDI-MS and reverse-phase LC followed by MALDI-MS have been used to delineate the BALF proteome of both healthy smokers and COPD patients [37]. In particular, Merkel et al. detected in BALF from smokers with COPD, in comparison with asymptomatic smokers, higher concentrations of lysozyme C, calgranulins A/B, and neutrophil defensins 1/2 [21]. Moreover, Plymoth et al. demonstrated that when baseline BALF proteome is profiled in apparently healthy smokers, compared to nonsmoker controls, and then reevaluated after 6-7 years, it is possible to find different protein signatures in those subjects who will develop GOLD stage 2 COPD [22]. Therefore, these differences can be used as predictor biomarkers of smokers' susceptibility to develop COPD through its progressive severity stages. Furthermore, Pastor et al. have recently shown that BALF protein content can be usefully analyzed by 2D-PAGE coupled to MALDI-TOF/TOF in order to identify specific proteomic profiles that can help to distinguish COPD patients from subjects with lung cancer [38]. In particular, a total of about 40 proteins were differentially expressed in these two diseases, mainly related to biological networks involved in inflammatory signaling, free radical scavenging, oxidative stress response, and glycolysis and gluconeogenesis pathways.

## 7. Lung Tissues

Availability of lung tissues requires very invasive procedures such as endoscopy or surgery, necessarily restricted to research purposes and not applicable to daily clinical setting in real life. With regard to proteomic studies, the main limitation of this approach concerns the small numbers of lung specimens sampled, when related to the overall population of COPD patients. However, highly valuable information can be obtained by profiling protein expression in a real biological context directly reflecting lung pathology. By analogy with IS and EBC, for example, high levels of surfactant protein A were also detected in COPD lung, but not in normal and fibrotic lungs [23]. Using MALDI-TOF-MS analysis, Lee et al. showed that the expression of matrix metalloproteinase-13 (MMP-13) and thioredoxin-like 2 was increased in COPD lung tissues [24]. MMP-13, a proteolytic enzyme implicated in tissue damage and remodeling, was predominantly expressed in alveolar macrophages and type II pneumocytes. Thioredoxin-like 2 is an antioxidant protein whose expression was mainly located within the bronchial epithelium, largely and directly exposed to cigarette smoke and airborne pollutants. Therefore, thioredoxin-like 2 synthesis could be induced by the oxidative burden that plagues smokers' airways. Moreover, Kelsen et al. used a proteomic strategy based on 2-DE coupled with MALDI-TOF-TOF to study human lung tissue samples obtained from three groups of subjects, including nonsmokers, chronic smokers, and ex-smokers, respectively [25]. In persistent smokers, but not in the other two study groups, these authors observed an upregulation of the so-called “unfolded protein response,” consisting of a complex molecular cascade activated by cellular stressors such as highly toxic reactive oxygen and nitrogen species, which impairs protein folding inside the endoplasmic reticulum [39, 40]. The consequent compensatory response leads to overexpression of several different proteins involved in inflammation, apoptosis, energy metabolism, and cell cycle regulation [41]. In particular, Kelsen et al. found in current smokers, when compared to nonsmokers and ex-smokers, high levels of antioxidant enzymes, glucose-regulated protein 78, and calreticulin, associated with downregulation of S100-A9/calgranulin C, a proinflammatory protein [25]. The unfolded protein response could thus represent a protective cellular mechanism activated by an injured lung attacked by oxidative and inflammatory stresses.

## 8. Potential Clinical and Therapeutic Implications of Proteomics/Peptidomics in COPD

The main aim of applying proteomics and peptidomics to biological samples obtained from COPD patients refers to the discovery of potential diagnostic and prognostic biomarkers, hopefully useful to characterize different pathological phenotypes as well as unravel new underlying molecular pathways and therapeutic targets [42]. Within such a conceptual context, it is however very unlikely that a single biomarker can be sufficient for COPD diagnosis, monitoring, and phenotypic characterization. Rather, it is arguable that successful proteomic strategies should pursue the complex task of identifying coordinated panels of multiple biomarkers [43].  Table 1 summarizes the most important proteins/peptides detected by several proteomic/peptidomic methods in different biological samples obtained from COPD patients.

In particular, IS and EBC can be valuable sources of candidate COPD biomarkers, because these samples reflect the real airway biological environment and can be obtained by noninvasive procedures. Peptidomic profiling of IS is very attractive, given the crucial role played by peptides in many pathologic conditions, also including inflammatory and oxidative processes underlying the development and progression of COPD. Within this context, research efforts will be greatly facilitated by the continuous advances in standardization of protein fractionation techniques and mass spectrometric platforms, complemented by more efficient methods of peptide enrichment. In order to achieve the main goals of proteomic/peptidomic applications to COPD, it will be always very important to extend these studies to smokers and nonsmoking controls, thus pursuing in such a way the aim of discovering the cellular and molecular pathways responsible for individual susceptibility to this widespread disease.

Furthermore, the search for phenotype-specific biomarkers could help to better understand the individual driving mechanisms of disease as well as identify drug targets possibly useful for personalized treatments of COPD. However, the translational process consisting of information transfer from basic research to the real-life clinical setting is still in its infancy, and many years will be needed to perform larger investigations capable of validating and extending the utility for each COPD patient of the proteomic/peptidomic approaches.

## Figures and Tables

**Table 1:** Samples, MS methods, and major proteins/peptides (involved in COPD pathogenesis) detected.

Sample	Methods	Major proteins/peptides detected	Reference
Plasma	2D-PAGE^(a)^ MALDI-TOF	*α*_2_-Macroglobulin, haptoglobin, ceruloplasmin, and hemopexin	[7]

Serum	Microarray	Interleukin-8 and matrix metalloproteinase-9	[8]

Serum	SELDI-TOF	Serum amyloid A	[9]

Plasma	High-resolution LC/FT-ICR MS^(b)^	55 peptides mapped to 33 unique proteins, 12 of them with known roles in the complement or coagulation cascade	[10]

IS	2D-PAGE GeLC-MS/MS^(c)^	More than 250 proteins Three members of the annexin family, kallikreins 1 and 11, and peroxiredoxins 1, 2, and 5	[13]

IS	2-DE LC-MS/MS	Lipocalin and apolipoprotein A1	[14]

IS	2D-DIGE^(d)^ MALDI-TOF	Polymeric immunoglobulin receptor (PIGR)	[15]

IS	CapLC-MS/MS	203 proteins identified, Zn-*α*_2_-glycoprotein, Clara cell, PRR4, LPLUNC2 *β*-actin, MUC5A/C, MUC5B, histone 4, lipocalin 1, SPLUNC1, cathepsin G, Ig J, Ig *μ* heavy, and Ig *γ*1	[16]

IS	SELDI-TOF	Calgranulins and Clara cell secretory protein	[17]

IS	MSB^(e)^/MALDI-TOF	Antimicrobial peptides *α*-defensins	[18]

EBC	1-DE, 2-DE, *μ*-HPLC and ESI-MS/MS, and SELDI-TOF	Cytokines (IFN-*γ*, IL-2, and IL-15) Cytokeratins (CK-1, CK-9, and CK-10)	[19]

EBC	LC-MS/MS and SELDI-TOF	Cytokines, type I and II, cytokeratins, two surfactant protein A isoforms, and calgranulins A and B	[20]

BALF	SELDI-TOF RP-LC^(f)^ MALDI-TOF LC-MS/MS	Lysozyme C, calgranulins A and B, and neutrophil defensins 1 and 2	[21]

BALF	2D-PAGE MALDI-TOF	200 proteins in the BAL proteome, proteins associated with redox reactions, immune reactivity, and inflammation	[22]

Lung tissue	2D-PAGE MALDI-TOF	Surfactant protein A	[23]

Lung tissue	2D-PAGE MALDI-TOF	Matrix metalloproteinase-13 (MMP-13) and thioredoxin-like 2	[24]

Lung tissue	2D-PAGE MALDI-TOF	Antioxidant enzymes, glucose-regulated protein 78, calreticulin, and S100A9/calgranulin C	[25]

IS: induced sputum. EBC: exhaled breath condensate. BALF: bronchoalveolar lavage fluid.

^(a)^2D-PAGE: two-dimensional polyacrylamide gel electrophoresis.

^(b)^FT-ICR MS: Fourier transform-ion cyclotron resonance.

^(c)^GeLC-MS/MS: proteins are separated by standard SDS-PAGE gel which is then cut into 25 pieces. Each gel slice is then cleaved by trypsin and then further fractionated by LC separation. Peptide fragments are then analysed by MS/MS.

^(d)^2D-DIGE: two-dimensional difference gel electrophoresis.

^(e)^MSBs: mesoporous silica beads.

^(f)^RP-LC: reverse-phase liquid chromatography.
